# Mapping the landscape of AI in healthcare in Kazakhstan: a scoping review of readiness, development, and adoption

**DOI:** 10.1186/s12913-026-14484-4

**Published:** 2026-04-04

**Authors:** Shnara Svetlanova, Indira Karibayeva, Sholpan Aliyeva, Valikhan Akhmetov, Bekdaulet Akimniyazova, Nazgul Shubatkaliyeva, Nadira Aitambayeva, Laila Nazarova, Nazerke Narymbayeva, Ainur Qumar

**Affiliations:** 1 Department of Health Policy and Management, Asfendiyarov Kazakh National Medical University, Almaty, Kazakhstan; 2 Department of Health Policy and Community Health, Jiann-Ping Hsu College of Public Health, Georgia Southern University, Statesboro, USA; 3 National Hospital of the Medical Center of the Presidential Administration, Almaty, Kazakhstan; 4 Kazakhstan Medical University, Almaty, Kazakhstan; 5 Erensau Hospital, Almaty, Kazakhstan

**Keywords:** Artificial intelligence, Machine learning, Healthcare, Kazakhstan, Digital health, AI readiness, AI implementation, Scoping review

## Abstract

**Background:**

The rapid development of artificial intelligence (AI) and machine learning (ML) technologies has created new opportunities for improving healthcare systems worldwide. In Kazakhstan, national digitalization initiatives have supported the introduction of electronic health records and medical information systems; however, the level of AI readiness, development, and real-world implementation in healthcare remains insufficiently explored.

**Objective:**

This study aimed to synthesize current evidence on the readiness, development, and implementation of AI and ML technologies in the healthcare sector of Kazakhstan.

**Methods:**

A scoping review was conducted following the Arksey and O’Malley methodological framework and reported according to the PRISMA-ScR guidelines. The protocol of this scoping review was registered on the Open Science Framework (OSF). Electronic searches were performed in PubMed, Scopus, Web of Science, ScienceDirect, and Google Scholar for studies published between 2020 and 2025. Inclusion criteria covered empirical studies, policy analyses, and mixed-methods research focusing on AI in Kazakhstan’s healthcare context. Data were extracted using a standardized template and synthesized across three domains: AI readiness, AI development, and AI implementation.

**Results:**

A total of ten studies were included in the final synthesis. Evidence of AI readiness was mainly reported at the educational, professional, regulatory, and system levels, with limited workforce preparedness, insufficient formal training, and gaps in data protection and ethical regulation. AI development was primarily concentrated on technical model creation, including deep learning for medical imaging and automated laboratory interpretation, but often lacked clinical validation. Real-world AI implementation was reported in a small number of clinical settings, particularly in rehabilitation and laboratory medicine, where AI tools were reported to improve workflow efficiency, diagnostic support, and documentation processes.

**Conclusions:**

The Kazakhstan AI/ML healthcare literature is emerging and heterogeneous. While technical development and foundational digital infrastructure are advancing, evidence of routine clinical implementation remains limited, and readiness gaps persist in training, governance, interoperability, and regulatory oversight. Future research should prioritize implementation-focused evaluations, clinical validation, and governance models to support safe adoption across healthcare settings.

**Clinical trial number:**

Not applicable.

**Supplementary Information:**

The online version contains supplementary material available at 10.1186/s12913-026-14484-4.

## Introduction

Artificial intelligence (AI) in healthcare is currently applied across major domains including clinical decision support, medical imaging and diagnostics, patient monitoring and personalized care, as well as health system management, reflecting its growing role in both patient-level and system-level innovation [1]. AI-powered chatbots have been shown to reduce repeat hospitalizations, improve patient engagement, and decrease waiting times for consultations in several healthcare settings [1, 2]. They are widely used across both high-income countries (HICs) and low- and middle-income countries (LMICs) for managing chronic diseases, providing psychological support, and educating patients, and have been shown to be effective [2].

The use of AI in everyday life, as well as in medicine, does not mean that it will completely replace medical workers, but rather that it will lead to the development of medicine and close collaboration between AI technologies and qualified medical professionals. In other words, technology will help medical professionals reduce the amount of routine work they do [3].

When implemented correctly in healthcare, AI can be economically beneficial, enabling personalised treatment methods, saving time and performing everyday tasks [4]. For the proper formation and implementation of AI in the healthcare sector, this can be achieved by reforming this area, as well as by searching for a specific way to implement AI [5].

In addition, medical workers play a key role in the healthcare sector. Currently, healthcare professionals in the healthcare sector are positive about the use of AI, provided they have undergone a certain training programme [6]. It is believed that the resolution of ethical and technological issues will be determined by the development of normative legal acts on the use of AI in healthcare [7].

For AI to be used to its full potential, it is important that medical workers understand the algorithmic logic, types of data and system limitations of AI. Therefore, it is necessary to implement AI training programmes for staff, as well as training in digital communication [8]. It is crucial for medical professionals to be able to identify ethical and legal issues when implementing and using AI tools. Understanding these aspects will enable them to use AI correctly in healthcare [9].

The utilisation of AI in the medical field has already demonstrated its efficacy; however, its implementation and integration into the daily operations of medical organisations continue to present challenges. Research in this field has historically focused on the capabilities of technologies rather than on how these systems are practically implemented in everyday clinical practice. It is therefore important to study the AI implementation process more closely in order to make it more understandable, effective, and sustainable [10].

Despite the active development and implementation of AI technologies in healthcare in many countries, existing research is primarily focused on high-income countries and specific clinical areas. Meanwhile, data on the preparedness of healthcare systems, the level of AI integration and the acceptance of these technologies in Central Asian countries remains limited and fragmented. In particular, there is a lack of systematic information on the current state of development, application and organisational conditions for the implementation of AI in the healthcare system in Kazakhstan.

Therefore, this scoping review aimed to synthesize current evidence on the readiness, development, and implementation of AI and ML technologies in the healthcare sector of Kazakhstan.

## Materials and methods

### Study design

This study adopted a scoping review methodology in accordance with the framework proposed by Arksey and O’Malley and was reported following the PRISMA-ScR (Preferred Reporting Items for Systematic Reviews and Meta-Analyses Extension for Scoping Reviews) guidelines [11]. The PRISMA-ScR checklist was used to guide the reporting of the review process. A scoping review approach was selected to systematically map the existing evidence on the development, implementation, effectiveness, and readiness of AI and ML technologies in the healthcare sector of Kazakhstan. Previous research indicates that real-world implementation of AI in healthcare remains insufficiently studied, particularly in emerging healthcare systems. Therefore, a scoping review was considered the most appropriate method to explore the extent, range, and nature of research activity in this evolving field. This methodology enabled the identification of key themes, research gaps, and patterns in the current literature without restricting the review to specific study designs or outcome measures. The review process followed the key stages of the Arksey and O’Malley framework, including identifying the research questions, identifying relevant studies, study selection, charting the data, and collating and summarizing the results.

### Identifying the research questions

In accordance with the Arksey and O’Malley framework for scoping reviews, the first step of the review process involves clearly defining the research questions. To address the aim of this review, the following research questions were formulated:

1. What is the level of AI readiness in Kazakhstan’s healthcare sector?
2. What evidence exists regarding the development of AI technologies in Kazakhstan’s healthcare system?
3. What evidence exists regarding the implementation of AI in clinical practice in Kazakhstan?

### Conceptual framework

To ensure conceptual clarity, this review was guided by an implementation-oriented systems framework in which AI adoption in healthcare is conceptualized as a staged yet interdependent process consisting of three analytically distinct domains: readiness, development, and implementation.

Within this framework, readiness refers to the structural, regulatory, workforce, and infrastructural conditions that enable or constrain the adoption of AI technologies. Development refers to the technical creation, training, and validation of AI models and systems intended for healthcare use. Implementation refers to the integration of these tools into real-world clinical or organizational workflows.

Distinguishing between these domains is particularly important in rapidly digitalizing healthcare systems, where the presence of digital infrastructure or AI prototypes does not necessarily indicate routine clinical implementation.

**Fig. 1:**
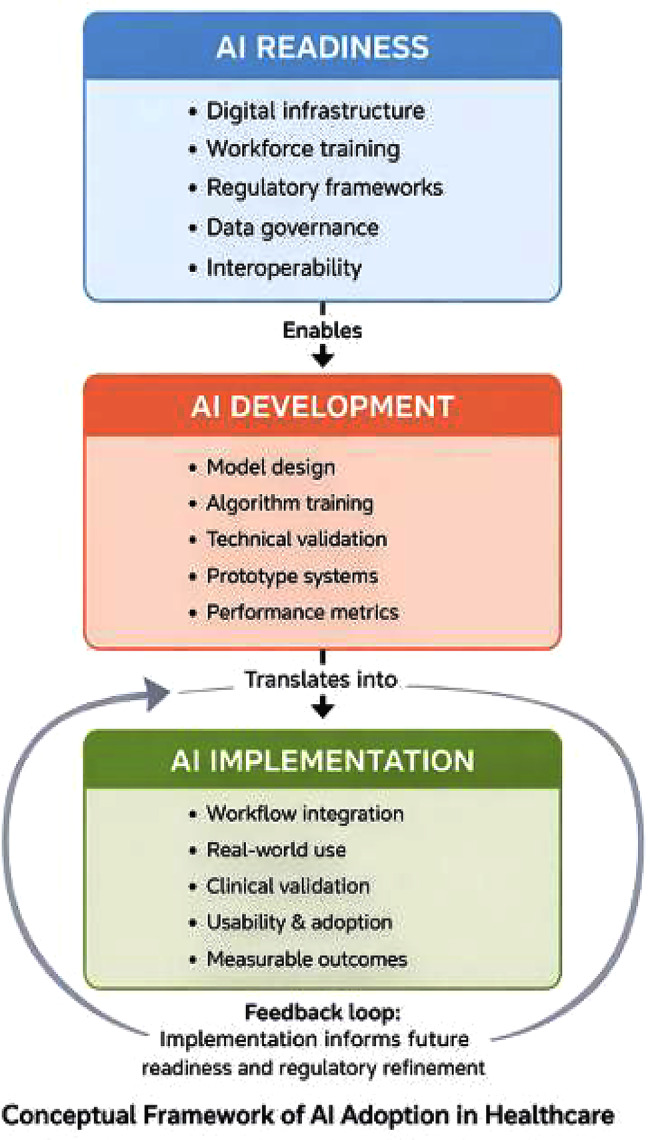
Conceptual framework of AI adoption in healthcare

This framework guided the classification of studies and the thematic synthesis of findings in the present review (Fig. 1).

### Study context

The evolution of AI in Kazakhstan’s healthcare sector is closely linked to the broader process of national digital health transformation. Over the past decade, Kazakhstan has implemented a series of legislative, infrastructural, and technological reforms that have progressively established the digital foundation necessary for future AI integration.

As shown in Table 1, the process began with the 2013 Concept for the Development of eHealth [12] which defined the strategic direction for healthcare digitalization, including the introduction of medical information systems (MIS), electronic prescriptions, and integration with national health databases. This policy marked the transition from paper-based to digital healthcare management.

**Table 1:** Key milestones in the digital transformation of healthcare in Kazakhstan

Legislative change	Year	What was introduced		Practical changes
Concept for the development of e-health in Kazakhstan [12]	2013	The foundations for the digitalisation of healthcare have been laid: implementation of MIS, electronic prescriptions, integration with national databases		The implementation of MIS, electronic medical records and digital services has begun.
Law of the Republic of Kazakhstan ‘On Informatisation’ № 418-V ZRK [13]	2015	Requirements for national information security and software certification established		MIS that have passed verification are included in the trusted software registry.
Implementation of electronic medical records (EMR) [14]	2015–2023	The transition from paper cards to digital cards		All medical organisations operate in MIS and Ministry of Health systems.
Integration of MIS with state registers	2023	Connection to the attached population register, resource management, vaccination		Double entry of data is excluded
Digitisation of vaccination [15]	2023	All vaccination data is entered into the MIS.		The information is automatically transferred to the Ministry of Health system.
Digital registration of pregnant women and women of childbearing age [16]	2024	Data transfer to the RBZhFV 2.0 system has been implemented.		Improved reproductive health monitoring
Electronic prescriptions [17]	2023–2024	Prescribing medicines via MIS		Reduction of errors and paperwork
Digitisation of medical certificates [18]	2023–2024	Forms 073/u, 075/u, 026/u, etc. have been converted to electronic format.		Simplified access to documents
Ministry of AI and Digital Development [19]	2025		Establishment of a dedicated national body to oversee AI strategy, coordination, and integration across sectors, including healthcare	Formal governance structure for AI policy; cross-sector AI coordination initiated; support for AI pilot projects in healthcare and development of national AI roadmap

The 2015 Law on Informatization [13] further strengthened the digital framework by introducing national standards for information security and certification of health information systems. As a result, verified MIS platforms were incorporated into the national registry of trusted software, ensuring system reliability and data protection.

Between 2015 and 2023, the large-scale implementation of electronic medical records (EMRs) enabled healthcare organizations to shift from paper documentation to fully digital patient records. This period also saw progressive integration of MIS with national registries, including population attachment databases, vaccination registries, and healthcare resource management systems. These integrations reduced data duplication and improved continuity of care.

In 2023, digitalization expanded to public health monitoring, including nationwide electronic vaccination tracking and automatic data exchange with Ministry of Health systems [15]. Subsequent developments in 2024 introduced digital registries for pregnant women and women of reproductive age, strengthening maternal and reproductive health surveillance [16].

At the clinical service level, the introduction of electronic prescriptions (2023–2024) [17] reduced medication errors and administrative workload, while the digitalization of standardized medical certificates simplified access to official health documentation for both patients and providers.

These reforms significantly strengthened Kazakhstan’s digital health infrastructure and created the technical prerequisites for AI applications.

### Identifying relevant studies

The search strategy was developed to identify studies addressing AI and ML technologies in the healthcare sector of Kazakhstan. Three core concepts guided the search process: AI, healthcare, and implementation/readiness (Table 2). Synonyms for each concept were iteratively tested to ensure comprehensive coverage of relevant literature.

**Table 2:** Concept areas and synonyms used to develop the search strategy

Search concepts (combined using “AND”):
Artificial Intelligence
Healthcare
Implementation/Readiness
Search terms (combined using “OR”):
Artificial Intelligence:
Artificial intelligence, Machine learning, Deep learning, Neural networks
Healthcare: Healthcare, Health care, Medical care, Clinical practice
Implementation/Readiness:
Implementation, Adoption, Readiness, Development, Integration

For clarity, AI was conceptualized at three levels:

1. AI algorithms and models (e.g., neural networks, deep learning),
2. AI applications (e.g., diagnostic or decision-support tools), and
3. AI systems (AI applications implemented within real healthcare contexts).

Implementation was defined as an intentional effort to integrate AI-based interventions into routine healthcare practice, including clinical, organizational, and system-level use. Search terms within each concept area were combined using the Boolean operator OR, and the three concept areas were linked using AND. The complete search strategy is provided in Multimedia Appendix 1. To capture both general and health-specific literature, five electronic databases were independently searched by two researchers (Sh.S. and I.K.): PubMed, Scopus, Web of Science, ScienceDirect, and Google Scholar.

### Eligibility criteria

The review incorporated original empirical studies on the application, implementation, evaluation, or perception of AI and ML technologies in healthcare. The present study exclusively encompasses research conducted in Kazakhstan or analysing the Kazakhstani healthcare system context. A comprehensive range of research designs was encompassed in the study, including quantitative, qualitative, mixed methods, ML analytics, clinical and diagnostic studies. Additionally, comparative policy and management analyses were incorporated to reflect systemic and regulatory readiness. A comprehensive selection of full-text articles in English, Russian and Kazakh was made available for review. No publication date restrictions were used.

Studies not related to healthcare were excluded from the analysis, as were AI studies not related to Kazakhstan. The following publications were excluded from the study: book chapters, conference materials and theoretical publications lacking empirical data. In the course of the study, literature on digitalisation that did not make use of AI or ML, as well as articles that were not fully accessible, were also excluded from consideration.

### Study selection

All identified records were exported into Mendeley reference management system, and duplicate entries were removed. The titles and abstracts of the remaining records were screened for eligibility in accordance with the predefined inclusion and exclusion criteria. Full-text articles were subsequently retrieved and assessed for eligibility. Studies were excluded if they did not address AI or ML in the healthcare context of Kazakhstan, lacked relevant outcomes related to development, implementation, effectiveness, or readiness, or failed to meet the eligibility criteria. Two researchers independently performed the study selection process (Sh.S. and I.K.). Two lists were then compared and merged. Any uncertainties regarding study inclusion were resolved through discussion and consultation with the third author (V.A.).

### Charting the data

A standardized data extraction template was developed to systematically chart information relevant to the research questions. The conceptualization of AI was guided by the World Health Organization’s definition, which describes AI as a machine-based system capable of making predictions, recommendations, or decisions that influence real or virtual environments with varying levels of autonomy [20]. For each included study, the following data were extracted:

General information: Authors, year of publication, country, healthcare setting, study aim, and study design. AI development and application: Type of AI technology, AI model or method used (e.g., ML, deep learning, natural language processing), type of task performed (e.g., diagnosis, classification, interpretation), intended use of AI, and target users (e.g., clinicians, students, patients, health systems). Implementation and readiness: Research focus, level of readiness (educational, regulatory, organizational, or system-level), evidence of real-world implementation, reported outcomes (e.g., effectiveness, usability, efficiency), and identified barriers or facilitators.

Data extraction was performed systematically across all included studies to ensure consistency, comparability, and comprehensive coverage of the predefined thematic domains. Two researchers (Sh.S. and I.K.) independently extracted the data using standardized datasheets, which were subsequently compared and reconciled. Any discrepancies or uncertainties were resolved through discussion and, when necessary, consultation with a third author (V.A.), until a consensus exceeding 90% agreement was achieved.

### Risk of bias assessment

The methodological quality of included studies was assessed using the Mixed Methods Appraisal Tool (MMAT). The MMAT consists of two initial screening questions followed by five methodological criteria specific to each study design category (qualitative, randomized controlled trials, non-randomized studies, quantitative descriptive studies, and mixed methods). Each criterion is rated as “Yes,” “No,” or “Can’t tell.” In line with MMAT guidance, overall quality scores were not used as exclusion criteria; however, for descriptive purposes, the number of criteria met (out of five) was summarized to reflect study quality. The detailed results of the quality appraisal for each included study are presented in Supplementary Table 1.

### Summarizing the results

The extracted data were mapped and summarized in relation to the three main domains of the review: development, implementation, and readiness/effectiveness of AI technologies in healthcare. A qualitative thematic synthesis approach was used to analyze evidence related to implementation processes, readiness, and reported outcomes. Included articles were read repeatedly to identify key concepts and patterns. Initial codes were generated for each study and grouped into broader thematic categories, such as workforce readiness, regulatory environment, technical development, clinical integration, and system-level adoption. Themes were refined through comparison across studies to identify similarities, differences, and overarching patterns. The results were then organized into structured tables and narrative summaries to provide a comprehensive overview of the current state of AI in Kazakhstan’s healthcare sector. The synthesis was guided by a structured conceptual framework distinguishing three operational domains: readiness, development, and implementation. To ensure consistent classification across studies, explicit criteria were applied.

AI readiness was operationally defined as evidence describing the capacity of individuals, organizations, or systems to adopt AI technologies. This included workforce knowledge and training, regulatory and legal preparedness, digital infrastructure, governance structures, interoperability, and national-level digital maturity indicators.

AI development was defined as the technical creation, adaptation, or validation of AI models, algorithms, or systems intended for healthcare applications. Studies were classified under development if they primarily focused on model design, technical performance metrics, algorithm training, data processing pipelines, or prototype systems, regardless of whether clinical deployment occurred.

AI implementation was defined as the deliberate integration of AI systems into routine healthcare practice or organizational workflows. Implementation required evidence of real-world use, clinician or user interaction, workflow integration, or measurable outcomes such as usability, efficiency, clinical accuracy in practice, or organizational impact.

Each included study was classified according to its primary analytic focus. Where studies addressed more than one domain, both domains were described.

## Results

### Search results

A total of 211 records were identified through the search process, including 207 records from electronic databases (PubMed, ScienceDirect, Scopus, Google Scholar, and Web of Science) and 4 additional records from official government websites. After the removal of 36 duplicate records, 171 records remained for title and abstract screening. During this stage, 136 records (79.5%) were excluded for not meeting the inclusion criteria.

**Fig. 2:**
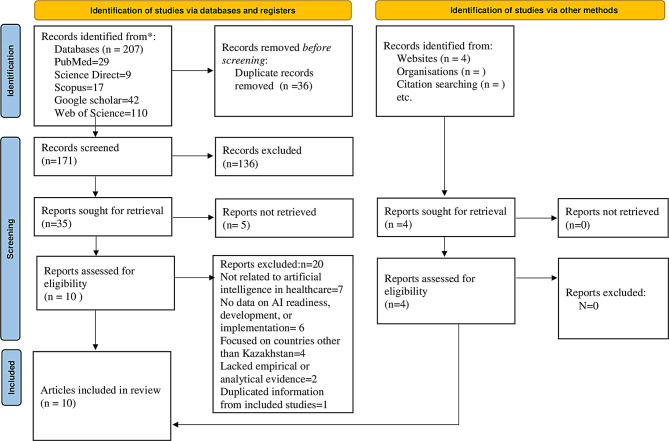
PRISMA-ScR flow diagram of study selection

The full texts of 35 articles (20.5%) were sought for retrieval. Of these, 5 articles could not be accessed, leaving 30 full-text articles for eligibility assessment. Following full-text screening, 20 articles (66.7%) were excluded due to reasons such as lack of relevance to AI in healthcare, absence of data on AI readiness, development or implementation, focus on countries other than Kazakhstan, or insufficient empirical evidence. A number of articles were excluded during the full-text stage of the review process, as they did not align with the focus of this particular investigation. One study developed a ML model to predict ICU admission in pregnant women with COVID-19, but it focused exclusively on clinical prediction and did not address AI implementation or healthcare system readiness in Kazakhstan [21]. Another study analysed trends in the epidemiology workforce using predictive statistical methods, but it did not involve AI or ML in healthcare practice [22]. In addition, one conference study described machine learning algorithms for predicting cardiovascular disease; however, it did not focus on AI implementation or healthcare system readiness in Kazakhstan [23]. Finally, one publication examined AI adoption in human resource management within corporate projects, which is outside the healthcare scope of this review [24]. Finally, 10 studies (33.3%) met all eligibility criteria and were included in the scoping review. The study selection process is illustrated in the PRISMA-ScR flow diagram (Fig. 2).

### Study characteristics

Most of the included studies were published recently, between 2023 and 2025, reflecting the growing but still emerging interest in AI and ML in Kazakhstan’s healthcare sector. This temporal distribution is consistent with the relatively recent national digital health initiatives and policy developments supporting AI adoption. The majority of the reviewed studies were empirical in nature, including cross-sectional surveys, qualitative interviews, analytical ML studies, and a randomized controlled trial. However, a substantial proportion of the literature consisted of policy analyses, methodological papers, and review-based studies, indicating that AI implementation in Kazakhstan is still largely discussed at a conceptual and exploratory level rather than being widely evaluated through large-scale clinical trials. The results are presented according to the predefined conceptual framework distinguishing readiness, development, and implementation domains, enabling structured interpretation of Kazakhstan’s AI ecosystem in healthcare.

### Research question 1: AI readiness in Kazakhstan’s healthcare sector

Seven studies examined different aspects of readiness for AI and ML in Kazakhstan’s healthcare system, including educational, professional, regulatory, scientific, and national-level readiness (Table 3). Educational readiness among future healthcare professionals was generally low. In a cross-sectional survey of 127 medical students, 95.3% reported having no formal AI education, with an average knowledge score of 32.65 out of 100 and a low readiness score (MAIRS-MS 2.64 ± 0.68) [27]. Similar findings were reported among 213 dental students, where 66.7% had basic knowledge of AI, but only 40.9% were aware of its application in dentistry [30]. These results indicate limited preparedness of the future workforce for AI-based healthcare.

**Table 3:** AI readiness

Authors, year	Design	Selection/sources	Level of readiness	Tools/methods	Quantitative data	Main barriers	Conclusion
Ahmad et al., 2025 [25]	ML analysis	1960–2020 data	National	PCA, K-means, Decision Tree	Accuracy 69%, Kazakhstan – Transition Economy	Economic constraints	Moderate readiness
Baymurza et al., 2025 [26]	Overview + survey	n = 41	Scientific	SWOT analysis	Positive attitude, low awareness	Shortage of specialists, poor awareness	Early-stage genomics
Cruz et al., 2025 [27]	Cross-sectional quantitative	n = 127 medical students	Educational, personnel	MAIRS-MS, GAAIS	95.3% without training; knowledge 32.65/100; MAIRS-MS 2.64 ± 0.68	Lack of education, poor understanding of AI	Low readiness of future specialists
Qumar et al., 2024 [28]	Overview	Healthcare system	Systemic	Policy analysis	20+ MIS, EMR, e-prescriptions	Fragmentation, digital inequality	High digitalisation, AI partially
Tazhiyeva & Mytalyapova, 2025 [29]	Analytical	Legislation	Regulatory	Content analysis	No quantitative data available	Gaps in data protection, ethics	Regulation is not ready
Yasa et al., 2025 [30]	Cross-sectional quantitative	n = 213 dentists	Educational	Online survey, χ^2^	66.7% are familiar with AI; 40.9% are familiar with AI in dentistry	Lack of courses, low awareness	Readiness is limited by knowledge
Zhaksylykova et al., 2025 [31]	High quality	n = 15 healthcare workers	Professional	Semi-structured interviews	No quantitative data available	Ethics, responsibility, cybersecurity	Training and regulation required

Professional readiness was explored through qualitative interviews with 15 healthcare workers [31]. Participants expressed generally positive attitudes toward AI but raised concerns regarding ethical responsibility, data security, and accountability. The authors emphasized the need for structured training programs and regulatory guidance to support safe implementation. Scientific readiness for AI in genomic medicine was assessed through a mixed review and survey study (*n* = 41) [26]. Although respondents showed a positive attitude toward AI, awareness of current genomic AI applications remained low. Key barriers included a shortage of trained specialists and limited public awareness, suggesting that AI in genomics remains at an early research stage in Kazakhstan.

Regulatory readiness was evaluated through a legislative analysis [29]. Significant gaps were identified in personal data protection, ethical regulation, and coordination between legal frameworks. No empirical evidence of AI implementation was reported, indicating that regulatory preparedness remains insufficient.

At the national level, a ML-based analysis using PCA, K-means clustering, and decision tree models classified Kazakhstan as a “transition economy” with moderate digital readiness [25]. The decision tree model achieved an accuracy of 69%, showing that economic indicators alone do not fully explain digital adaptation.

System-level digital readiness was described in a national policy review, which reported widespread implementation of medical information systems (MIS), electronic medical records, and e-prescriptions, with more than 20 registered MIS platforms in use [28]. However, challenges such as system fragmentation, digital inequality, and limited interoperability were noted. While digital infrastructure was well developed, AI implementation remained partial.

Overall, readiness for AI in Kazakhstan’s healthcare sector was found to be uneven, with stronger digital infrastructure but limited educational, regulatory, and workforce preparedness.

### Research question 2: AI development in Kazakhstan

Four studies reported on the development of AI technologies in healthcare (Table 4). One deep learning study developed a convolutional neural network (CNN) and EfficientNetB0 model for automated classification of diabetic retinopathy using 35,126 training images and 3,662 test images [32]. The model achieved approximately 80% accuracy, with weighted precision, recall, and F1-scores above 0.80. Advanced techniques such as data augmentation, cross-validation, transfer learning, and fine-tuning were applied to improve performance. However, no clinical validation, regulatory approval, or system integration was reported, indicating that development remained at a technical stage.

**Table 4:** AI development

Baymurza et al., 2025 [26]	Overview	SWOT, survey	Genomic data	No clinical metrics	AI tools (descriptive)	No implementation	Research
Kadirkulov et al., 2023 [32]	Implementation	smartLAB + OpenAI	Laboratory data	Self-interpretation	HL7, OpenAI API	API dependency	Operational
Kalimoldayev et al., 2018 [33]	Methodology	Strategy analysis	Documents	No metrics	Theoretical models	No clinical data available	Political
Yesmukhamedov et al. 2025 [34]	DL model	CNN, EfficientNetB0	35,126 train/3,662 test	Accuracy ≈80%, F1 > 0.80	TensorFlow, Transfer Learning	No clinical validation	Technical

In the genomic medicine field, a review and SWOT-based analysis highlighted Kazakhstan’s scientific infrastructure and research potential for AI-based genomic analysis [26]. Despite positive attitudes, the absence of clinical AI tools and limited specialist availability suggested that development was still at a research and exploratory level.

A laboratory medicine study described the integration of the smartLAB laboratory information system with OpenAI for automated interpretation of laboratory results [32]. The system used HL7 standards and OpenAI’s API to generate clinical explanations, which were verified by laboratory physicians. This represented an operational-level development with real-time implementation, although dependency on external APIs was identified as a limitation.

A policy-oriented methodological study analyzed Kazakhstan’s AI development strategy based on national documents [28]. The study proposed theoretical frameworks and emphasized interdisciplinary cooperation but did not report technical or clinical AI implementations.

Overall, AI development in Kazakhstan ranged from experimental deep learning models to operational laboratory systems, with most applications still lacking large-scale clinical validation.

### Research question 3: AI implementation in clinical practice

Three studies provided evidence on real-world implementation of AI systems in Kazakhstan (Table 5). A randomized controlled trial in rehabilitation settings evaluated the MedQuest AI-assisted mobile application among 185 patients [32]. The AI-based ICF mapping demonstrated high agreement with clinicians’ assessments (κ = 0.842), and physician usability was rated as excellent (SUS = 86.8). The intervention significantly reduced documentation time compared with paper-based workflows, indicating evidence of successful pilot-level clinical integration.

**Table 5:** AI implementation

Authors, year	Design	Region	Sample	Metrics	Results	Implementation status
Kadirkulov et al., 2023 [32]	Implementation	Laboratory	Lab patients	Self-interpretation	Reduction of errors	Used
Kurban et al., 2025 [35]	RCT	Rehabilitation	n = 185	κ=0.842, SUS = 86.8	Time reduction, high precision	Actual implementation
Qumar et al., 2024 [28]	Overview	National	The entire system	MIS, EMR	Digital services	Partial AI

In laboratory medicine, the smartLAB-OpenAI system was implemented for automated interpretation of laboratory results [33]. The system reduced human error, improved efficiency, and supported real-time clinical decision-making. This tool was reported to be in active use in laboratory workflows.

At the national system level, digital health tools such as MIS, electronic medical records, and e-prescriptions were routinely used across healthcare institutions [28]. However, AI-specific applications were described as partially implemented, with most AI use limited to pilot projects or specific clinical domains. Overall, only a small number of studies demonstrated real-world AI implementation, with the strongest evidence coming from rehabilitation and laboratory medicine.

Across the included studies, AI readiness in Kazakhstan was highest at the digital infrastructure level but limited in education, regulation, and workforce preparedness. AI development was mainly technical and experimental, with few clinically validated systems. Real-world implementation was demonstrated in only a small number of settings, primarily in rehabilitation and laboratory services. Evidence of effectiveness, such as improved efficiency and high usability, was reported in select clinical trials, but large-scale adoption remains limited.

### Types and applications of AI technology

The reviewed studies demonstrated that AI applications in Kazakhstan’s healthcare sector are primarily focused on technical development and decision-support functions rather than fully autonomous clinical systems. Most AI technologies were based on ML and deep learning models, including convolutional neural networks (CNNs) for image classification, natural language processing tools for laboratory result interpretation, and analytical models for national digital readiness assessment. The most common tasks performed by AI systems included disease classification, data interpretation, clinical documentation support, and workflow optimization.

AI applications were mainly used in rehabilitation medicine, laboratory diagnostics, dentistry, genomics, and informing national digital health policy. Only a small number of studies reported AI systems being integrated into real clinical workflows, with the strongest evidence coming from rehabilitation and laboratory medicine. Most AI tools were designed to support healthcare professionals, particularly physicians and laboratory specialists, rather than to replace clinical decision-making. This reflects a prevailing approach in Kazakhstan where AI is viewed as an assistive technology rather than an autonomous clinical agent. AI applications in Kazakhstan remain narrow in scope, focused on specific clinical or analytical tasks, with limited expansion into broader healthcare services such as primary care, mental health, or infectious disease management.

### Implementation process

Evidence on the implementation of AI systems in Kazakhstan was limited and mainly derived from a small number of empirical studies.

Successful implementation was reported in rehabilitation and laboratory medicine, where AI tools were integrated into existing workflows. These systems improved documentation efficiency, reduced human error, and achieved high user acceptance. Key enabling factors included compatibility with existing information systems, clinician involvement, and usability of AI interfaces.

However, most studies did not provide detailed descriptions of implementation processes, organizational strategies, or change management approaches. There was little discussion of structured implementation frameworks, governance models, or long-term sustainability planning.

Barriers to implementation included:

- Lack of trained personnel
- Ethical and legal uncertainties
- Data security concerns
- Limited interoperability between systems
- Absence of national AI governance frameworks

These findings suggest that while technical feasibility has been demonstrated, system-level implementation strategies remain underdeveloped.

## Discussion

From an implementation science perspective, and drawing on The Consolidated Framework for Implementation Research (CFIR) [36], the findings of this review suggest that while Kazakhstan has made notable progress in digital health infrastructure and AI-related technical development (intervention characteristics), the progression from technological development to real-world adoption remains incomplete. Key organizational and structural determinants-including organizational readiness for implementation, workforce training and competency (individuals involved), and governance and leadership mechanisms (inner setting)-are comparatively less mature, thereby constraining the effective integration of AI into routine healthcare practice. This finding aligns with observations from implementation science frameworks, which underscore that technological innovation alone is insufficient to ensure successful integration into routine healthcare practice. Without supportive regulatory structures, trained personnel, and implementation strategies, many promising technologies remain confined to pilot projects or experimental settings.

A more detailed mapping of the findings onto CFIR domains further highlights the structural nature of implementation challenges. Barriers related to limited clinical validation and variability of AI tools correspond to the “intervention characteristics” domain. Workforce-related gaps, including insufficient training and limited digital competencies, align with the “characteristics of individuals.” Organizational constraints, such as lack of institutional readiness and integration into workflows, reflect challenges within the “inner setting.” At the system level, regulatory gaps, governance limitations, and data infrastructure issues correspond to the “outer setting.” This multidimensional pattern indicates that AI implementation in Kazakhstan is constrained not by a single factor, but by the combined immaturity of several interdependent domains.

A review of the literature indicates that the level of readiness to utilise artificial intelligence in terms of human capital, encompassing medical personnel, exhibits a lag behind technological progress in both regulatory and managerial domains. In summary, the degree of training provided to medical personnel, in terms of both management and managerial skills, is inadequate. In the field of education, it has been observed that medical students receive only a rudimentary level of training in artificial intelligence [30]. An analysis of the regulatory framework has revealed gaps in the areas of ethical regulation, data protection and AI governance coordination. The findings of this study suggest that Kazakhstan demonstrates partial structural readiness for AI adoption, primarily driven by the expansion of digital health infrastructure. However, managerial capacity, regulatory coordination, and workforce preparedness remain less developed.

The reviewed studies indicate that AI development in Kazakhstan is primarily concentrated on technical and experimental solutions, including models for analyzing medical images and laboratory data. While these systems demonstrate promising technological performance, most remain at the stage of prototype development or limited pilot deployment. This suggests that technological capability is advancing faster than the mechanisms required for large-scale clinical validation and implementation.

The integration of artificial intelligence within the healthcare sector is regarded as a pivotal concern. However, research in this area is limited, with only a small proportion of studies examining the utilisation of artificial intelligence in the daily work of doctors in rehabilitation centres and laboratories [35]. The available evidence suggests that real-world implementation of AI in Kazakhstan remains limited and concentrated in a small number of clinical contexts, particularly rehabilitation and laboratory medicine. This pattern indicates that AI adoption currently occurs mainly at the level of pilot initiatives or localized deployments rather than widespread institutional integration across the healthcare system.

In the event that the healthcare system is not adequately prepared at the outset, even efficacious prototypes will present considerable challenges in terms of implementation. It is imperative to emphasise that the utilisation of technology in a clinical setting is contingent upon its successful completion of comprehensive clinical trials. The absence of such rigorous testing precludes the possibility of reliable and consistent practical application. In the absence of systematic evaluation of the implemented artificial intelligence systems, it is not possible to ascertain which systems are functioning correctly and which require enhancement. Consequently, the formulation of regulatory frameworks and the training of specialists will be protracted [37].

International experience indicates that the successful implementation of artificial intelligence depends not only on technological innovation, but also on governance structures, system interoperability, and user trust. Similar patterns have been observed in several upper-middle-income and transition economies, where the expansion of digital infrastructure has outpaced the regulatory and organizational capacity required for large-scale AI adoption [38, 39]. The findings of this review suggest that Kazakhstan may be experiencing a comparable trajectory [40].

The implementation of artificial intelligence in healthcare is contingent not only on technological capabilities, but also on human factors and the organisational structure of work. It is imperative that specialists possess the requisite confidence and understanding of artificial intelligence, and that they receive support from the system. In countries where the development of artificial intelligence is proceeding in a satisfactory manner, a regulatory framework is generally established, specialists are being trained, technologies are being tested and are gradually being implemented across the system. In Kazakhstan, however, technological development is outpacing the evolution of its healthcare system, resulting in a shortage of trained professionals and the absence of effective management structures [40].

There is still limited evidence of AI use in several key areas of healthcare, including primary care, mental health, infectious disease management, and public health. This suggests that current AI applications in Kazakhstan remain limited in scope and focused on specific clinical or analytical tasks rather than being integrated across the broader healthcare system [41].

The key challenge in Kazakhstan does not appear to be the absence of AI technologies, but rather the limited maturity of the broader implementation environment. Although technological innovation and pilot initiatives are emerging, the organizational, regulatory, and workforce conditions required for large-scale and sustainable AI adoption remain underdeveloped.

The interpretation of these findings should also take into account the heterogeneity of the available evidence. The included studies differed considerably in their design, including policy analyses, exploratory machine learning studies, qualitative interviews, and one randomized controlled trial. These types of studies provide different levels of evidence. For example, the randomized controlled trial conducted in a rehabilitation setting provides stronger empirical support for real-world implementation and effectiveness than conceptual or policy-based analyses. Therefore, the conclusions of this review should be interpreted with caution, as the current evidence base remains limited and methodologically diverse. This heterogeneity was also reflected in the MMAT quality appraisal results, which indicated varying levels of methodological rigor across the included studies.

## Conclusion

This scoping review shows that Kazakhstan has established important digital health infrastructure that may support the future integration of artificial intelligence in healthcare. Nevertheless, the evolution of this system has been disparate. The development of artificial intelligence technologies and their subsequent evolution are well underway, yet the establishment of comprehensive guidelines and precise mechanisms for the training of specialists and the implementation of artificial intelligence remains a significant area of research. The use of artificial intelligence remains limited and is currently concentrated mainly in pilot initiatives and experimental applications.

At the same time, several structural and systemic factors may explain why large-scale implementation of artificial intelligence in Kazakhstan’s healthcare system remains limited. Despite ongoing digitalization initiatives, the country still lacks centralized computing infrastructure capable of processing large-scale medical datasets and supporting advanced AI development. In addition, limited access to high-performance computing resources and the absence of a mature digital ecosystem for AI development, validation, and commercialization constrain the transition from experimental models to routine clinical application. Fragmentation of medical information systems and insufficient interoperability between digital platforms further complicate the scaling of AI solutions across healthcare institutions.

In order for artificial intelligence to be successfully implemented in the healthcare sector, there is a necessity for a coordinated and comprehensive approach to development. This involves the training of specialists, the establishment of clear rules and requirements, the clinical testing of technologies, and the structured implementation of these technologies in clinical practice. This will facilitate the transition from the testing phase to the safe and widespread utilisation of artificial intelligence.

In the future, it will be important to conduct research on the use of artificial intelligence, to understand what works best, and to develop an AI management and evaluation system to gradually improve perception.

These findings reinforce the need for implementation-oriented research and system-level strategies to bridge the gap between technological innovation and real-world clinical adoption.

## Limitations and methodological considerations

This scoping review has a few clear limitations. Firstly, there simply aren’t many studies yet as AI in Kazakhstan’s healthcare system is still in its infancy, so the pool was small from the outset. The studies themselves varied widely in terms of design, focus and what they were actually measuring. This made it difficult to compare them directly. Most of the published literature focuses on aspects such as readiness, policy, or technical groundwork rather than actual long-term clinical use or real-world results. There is probably also some useful grey literature, such as unpublished government pilot projects or internal reports, that has been overlooked.

In addition, potential publication bias and language bias should be considered, as the review included only studies available in English, Russian, and Kazakh and may not have captured unpublished pilot projects or grey literature related to AI implementation in Kazakhstan.

## Electronic supplementary material

Below is the link to the electronic supplementary material.

Supplementary material 1

## Data Availability

All data generated or analyzed during this study are included in this published article.

## Abbreviations

AI: Artificial intelligence
ML: Machine Learning
MIS: Medical Information Systems
CNN: Convolutional Neural Network
API: Application Programming Interface
EMR: Electronic Medical Record
