# Supplementary material for: Mapping the landscape of AI in healthcare in Kazakhstan: a scoping review of readiness, development, and adoption
Source: BMC Health Serv Res. 2026 Apr 4;26:611. doi: 10.1186/s12913-026-14484-4 (PMC13130796; doi:10.1186/s12913-026-14484-4)
Supplement: Supplementary file 1 — Supplementary material 1 [file 12913_2026_14484_MOESM1_ESM.docx]

**Supplementary Table 1**. MMAT risk of bias assessment results.

| **Author, year** | **Study type** | **S1** | **S2** | **Q1** | **Q2** | **Q3** | **Q4** | **Q5** |
| --- | --- | --- | --- | --- | --- | --- | --- | --- |
| Ahmad, 2025 | Quantitative (modeling) | yes | yes | yes | yes | yes | yes | yes |
| Baymurza, 2025 | Mixed methods | yes | yes | yes | partial | yes | no | yes |
| Cruz, 2025 | Quantitative descriptive | yes | yes | yes | no | yes | can’t tell | yes |
| Kadirkulov, 2023 | Implementation | yes | yes | yes | yes | partial | yes | yes |
| Kalimoldayev et al., 2018 | Policy / qualitative (document analysis) | yes | yes | yes | can’t tell | can’t tell | yes | no |
| Qumar, 2024 | Policy / descriptive | yes | yes | yes | yes | yes | yes | yes |
| Tazhiyeva, 2025 (29) | Qualitative | yes | yes | yes | yes | yes | yes | yes |
| Yasa, 2025 (26) | Quantitative descriptive | yes | yes | yes | no | yes | can’t tell | yes |
| Yesmukhamedov, 2025 | Quantitative (ML) | yes | yes | yes | yes | no | yes | yes |
| Zhaksylykova, 2025 | Qualitative | yes | yes | yes | yes | yes | yes | yes |
